# Do responsive sleep interventions impact mental health in mother/infant dyads compared to extinction interventions? A pilot study

**DOI:** 10.1007/s00737-022-01224-w

**Published:** 2022-04-05

**Authors:** Sarah Blunden, Joanne Osborne, Yaroslava King

**Affiliations:** Appleton Institute, Central Queensland University, 44 Greenhill Rd, South Australia Wayville, 5034 Australia

**Keywords:** Infant sleep, Controlled crying, Extinction, Stress, Depression

## Abstract

Methods to improve sleep in infants commonly involve some ignoring (extinction) but are often unpopular with mothers worried about infant distress when left to cry. Alternative more responsive methods are needed. This pilot study evaluated stress, maternal depressive symptomology and sleep in mother/infant dyads, between Responsive, Controlled Crying and Control groups. From 199 mother/infant dyads from any cultural background, 41 infants 4–12 months were randomly allocated to Responsive (RG, *n* = 15), Controlled Crying (CCG, *n* = 18) or Controls (Treatment as Usual, TAUG, *n* = 8), with 10 withdrawing after randomisation. Infant sleep (7-day sleep diaries) and stress (oral cortisol on two nights), maternal self-reported stress (Subjective Units of Distress, SUDS), maternal perceived infant distress (MPI-S) and symptoms of maternal depression (Edinburgh Post-natal Depression Scale, EPDS) were measured four times across 8 weeks. Sleep duration was not different between groups but Responsive woke less (*p* = .008). There were no differences in cortisol between groups across time points. Maternal SUDS was positively correlated with infant cortisol and MPI-S (*p* < *0.05)* and mothers in the Responsive group were significantly less stressed (*p* = *0.02)* and reported less symptoms of depression (*p* < *0.05)*. Findings in this small sample show Responsive methods are comparable to the extinction (Controlled Crying) in sleep outcomes but from a relational and maternal mental health perspective, are less stressful, offering families potential choices of sleep interventions.

## Introduction

Infants, especially in the first year of life, often require (Scher et al. 2005) parental assistance to initiate sleep and to reinitiate sleep after night wakings (Sadeh et al. 2010). Up to 40% of parents have reported that this sleep disturbance is concerning (Bruni et al. 2014; Honaker and Meltzer 2016).

Sleep fragmentation is consequential for mothers, with reported sleep loss, stress and exhaustion (Honaker et al. 2018). Maternal depression is a well-documented outcome of infant sleep disturbance (Hiscock 2010; Cook et al. 2012). Furthermore, maternal depression symptoms and infant sleep problems have bi-directional relationships (Dias and Figueiredo 2021) with maternal emotional distress (depressive, anxiety and parenting-stress symptoms) also predicting changes in infant sleep (Tikotzky et al. 2021). Infants with sleep disturbance cry more overnight (Hiscock 2010; Sadeh et al. 2016) , and maternal depression is strongly related to infant crying (Petzoldt 2018).

Detrimental child outcomes arising from problematic infant sleep include deficits in cognitive, emotional and behavioural functioning, but potentially, also detrimental effects on the mother’s perception of the relationship with their infant. This may reflect a disproportionate negative bias from parents around sleep disturbance in their infants and should alert health professionals to the level of stress this may cause to new parents (Meltzer et al. 2014).

Solutions to improve infant sleep disturbance usually include behavioural sleep interventions (BSI) (Blunden 2012) which are effective in ameliorating sleep and subsequently improving maternal well-being (Meltzer et al. 2014). The most commonly utilised BSI (Blunden et al. 2011) are based on the psychological concept of extinction. Extinction methods are based on behaviour theory’s principle of operant conditioning (Bouton 2007) where an unwanted behaviour (disruptive sleep and night-time crying) is ‘extinguished’ by ignoring that undesirable behaviours and eliminating the ‘reward’ component (parental attention) to encourage self-settling. All of extinction interventions withdraw parental assistance either immediately and completely (“cry it out”), more gradually (“controlled crying”) or very gradually (“camping out”) at sleep onset and overnight (Meltzer et al. 2014). However, in as much as the interventions are successful, they are dependent on parents having to ignore their crying child.

For several years, many parents have demonstrated reticence in undertaking extinction techniques (Etherton et al. 2016; Tse and Hall 2008) finding extinction methods unpalatable and too stressful, preferring to respond to their infant. In Australia, (Blunden and Bails 2013; Hiscock 2010) and Canada (Loutzenhiser et al. 2014), parents reported emotional and stress-related reasons in their reluctance to employ controlled crying. Whilst this reluctance may reflect differences in cultural or personal parenting ideologies, it has been reported that parental resistance revolves around fear about intense or extended crying being detrimental to the child’s health or fear of creating a poor attachment between parent and child (Maute and Perren 2018; Etherton et al. 2016). Four studies have reported no detrimental impacts of extinction on mother/infant dyads and to the contrary, mothers’ mental health (e.g. depression) improved (Gradisar et al. 2016; Cook et al. 2012; Bilgin and Wolke 2020; and poor sleep habits are avoided (Field 2017). Parents may be faced with increased depressive symptomology due to lack of sleep on the one hand, or high levels of stress when ignoring a crying infant to improve sleep on the other hand (Blunden and Dawson 2020).

Studies have attempted to objectively measure stress in infants who are left alone to self-settle measuring cortisol, which is an important indicator of stress and physiological arousal (Jansen et al. 2010). Cortisol measurement is difficult but regarded as important to measure infant stress levels during extinction BSI. Cortisol reactivity to stressors develops quickly throughout the first 12 months of life (Jansen et al. 2010) and elevated levels of cortisol in infants can be a result of an acute or chronic stressor. For acute stressors (such as inoculations), infant cortisol levels peak between 20 and 30 min post-acute stressor, returning to baseline cortisol levels 120–150 min post-stressor (Gunnar et al. 1996). This might suggest that 20–30 min into an extinction BSI, cortisol levels in distressed infants are likely to peak. The separation of mother and infant (aged 9–12 months) produces an increase in infant cortisol levels (Gunnar et al. 1992) and attentive maternal interaction impacts cortisol level recovery rates in older infants (Gunnar et al. 1996) but these are not always replicated (Gunnar et al. 2009). Furthermore, although cortisol levels are related to maternal responsiveness, they are not always aligned to the level of crying particularly in the early months (Gunnar et al. 1996). Both sleep and cortisol levels vary daily, so temporal relationships between 24-h sleep and cortisol regulation are still not well understood (Tuladhar et al. 2021).

As noted, efforts have been made to measure cortisol levels during extinction BSI. Gradisar et al. (2016) measured cortisol the morning after and Cook et al. (2012) the week after but neither reported higher stress levels in affected infants. However, cortisol was not measured at the time of separation (when parents feel the urge to comfort their child) but considerably later. Only Middlemiss et al. (2012) measured cortisol at the moment of separation in an extinction protocol, finding prolonged and elevated cortisol in infants in their sample. In another study, Middlemiss et al. (2017) reported actively responding and settling during BSI, showing no cortisol elevation. Similarly, Philbrook and Teti (2016) reported infants at 9 months had lower cortisol levels when their mothers were more emotionally responsive at sleep time.

Cortisol indicators therefore remain unclear at the moment of separation in extinction studies, yet clear when infants are not separated and soothed to sleep. There are to date no studies that have measured cortisol at the moment of separation in what could be termed a “responsive” BSI. Although definitions of “responsive” are not standardised (Paul et al. 2016), in this study, responsive interventions are defined as non-extinction based, methods that respond yet still teach an infant to self-settle by gradually reducing active settling (Blunden and Dawson 2020). Two studies have reported responsive methods significantly increased infant total night-time sleep and reduced overnight wakings, with high levels of parental satisfaction in soothing the crying infant (Blunden and Bails 2013; Boutzious et al. 2014).

The debate about stress levels during extinction BSI and evidence to show their efficacy (Meltzer et al. 2014) has done little to allay parental concerns about infant stress and may have underestimated a primary consideration — the powerful effects on a mother’s physiology, emotions and urge to reduce stress in their child, despite the instruction not to do so (Loman and Gunnar 2010). Indeed, the immediacy of this stress for parents seems to outweigh any benefits extinction BSI may offer, in which case the cure appears be worse than the problem itself (Blunden and Dawson 2020). It is necessary to better understand stress and depression levels during different types of BSI that utilise different levels of responsiveness. Measuring maternal stress levels objectively has been undertaken and may be beneficial (Gradisar et al. 2016; Middlemiss et al. 2012), but even subjective reports of their own stress and that of their infant can be informative. Measuring infant and maternal stress levels during a bedtime stressor, both objectively and subjectively *at the moment of the bedtime separation*, and estimations of maternal depression have not been undertaken in a *Responsive BSI* compared to an extinction protocol. This pilot study aimed to compare, for the first time in a randomised design, an alternative, Responsive method (gradual reductions in active comforting) to an extinction protocol (Controlled Crying) and a Control group, in ameliorating sleep disturbance including infant sleep duration overnight and the number of wakes after sleep inset (WASO), Objective and subjective differences in stress responses in the parent, the child and the mother/child dyad were compared in all groups. Additionally and importantly, the differences in maternal depression scores were measured across different levels of responsiveness.

## Method

### Participants

**Fig. 1:**
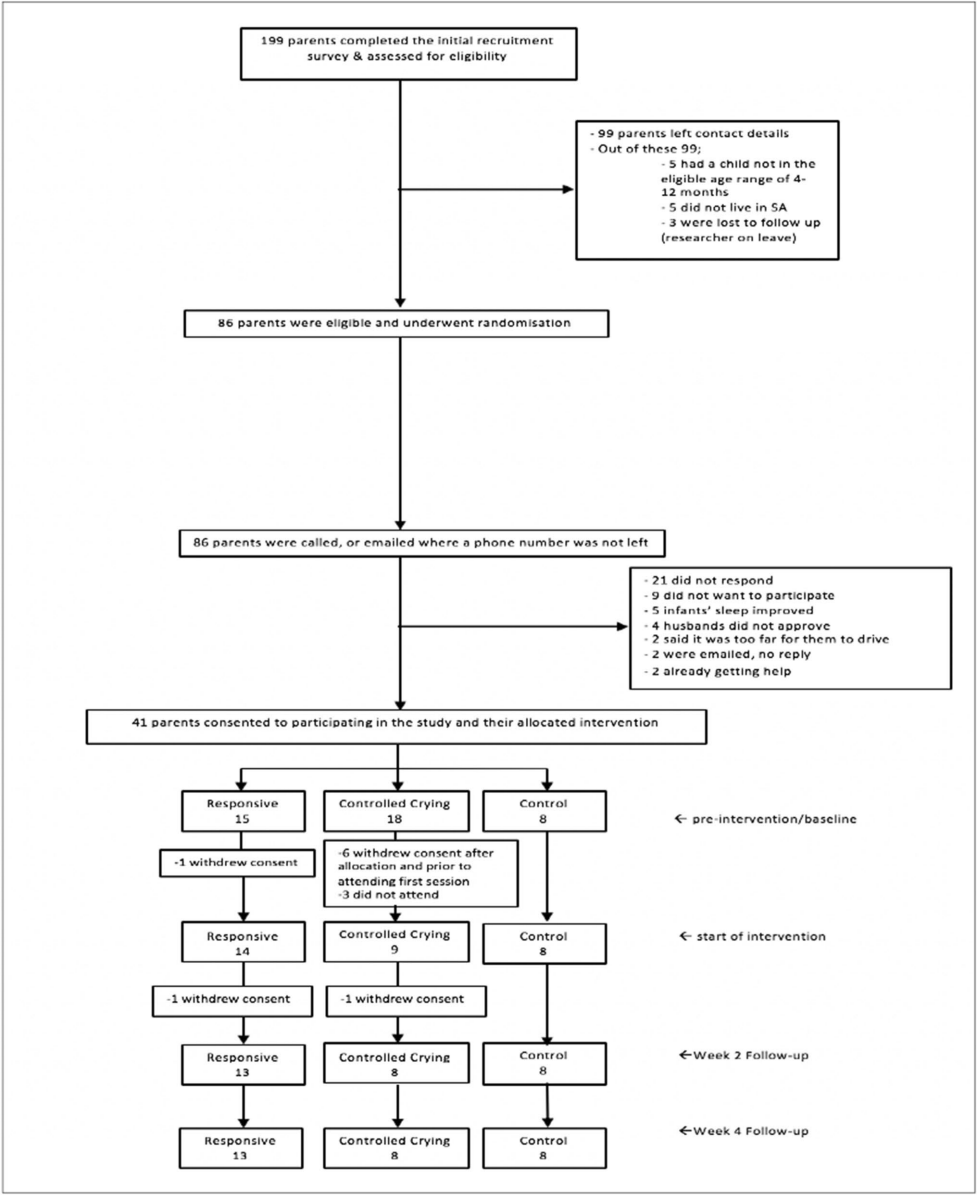
Visual representation of the study design and recruitment process

Between October 2016 and October 2017, an online survey targeted parents of children attending child care centres and specialty sleep and paediatric clinics in Adelaide, South Australia and via social media advertising. Information gathered included family demographic (family members’ age, gender, and primary language, birth order of infant, etc.) and sleep information. Infants aged 4–2 months, of English-speaking background residing in and met inclusion criteria of > 3 wakes per night, for > 4 days per week, nocturnal waking averaging more than 60 min or with < 9 h of total nocturnal sleep per night) (Sadeh 2004) were asked to join the research study investigating BSI in infants. Also based on Sadeh’s 2004 definition of “problematic infant sleep” from the parents’ perspective, parents rated their infant’s sleep as either no problem, or a “small” or “serious” problem. From 199 completed questionnaires, 41 were randomised with consent (using a rolling computer-generated algorithm) into three groups (Responsive, Controlled Crying and Controls). Ten families withdrew when allocated to a group they did not want and so the final sample was 31 mother/infant dyads (see Fig. 1).

- Responsive (R): Parents place their drowsy baby in their sleep space and sitting near their baby patting/stroking them to sleep. Over a few days, parent reduce patting/touching *to* sleep responding with verbal cues. Then parent moves gradually towards the door, can leave the room for short periods of time whilst awake, always responding with verbal cues, gradually extending the time between verbally responding and attending.
- Controlled crying (CC): Parents place their drowsy baby in their own sleep space, and then leave the room. If the baby starts to really cry, parents wait for 3 min before returning to briefly comfort the child but not pick them up. This continues adding 3 min to each interval before returning.
- Controls (C): Parents to do usual parenting settling, with intervention of their choice post study.

Participants who indicated wanting to change conditions were offered the opportunity to undertake an intervention of their choice post study. The study had ethical approval (H16/11–309) and was registered with Clinical Trials Registrar (xCTRN12617000428369) from 24/03/2017.

### Tools and measures

#### Subjective maternal distress

The Subjective Units of Distress Scale (SUDS) (Wolpe 1982; Kiyimba and O’Reilly 2020) is a 1-item 11-point Likert-type subjective anxiety scale, utilised for subjective stress and anxiety in a given moment, where 0 represents “Totally relaxed” and 100 “Highest distress you have ever felt”. It has demonstrated good convergent validity (strong correlations with anxiety scores), and good discriminant validity (discriminates between state vs trait anxiety) (Kim et al. 2008). The SUDS has been extensively used in the realm of behaviour treatment to measure subjective stress (Kim et al. 2008).

#### Maternal perception of infant distress (MPI-S)

Developed for this project, three items measured MPI-S. (1) Stress, “In your opinion, is your baby stressed?” Yes/No/Do not know, (2) Crying, “In the present moment is your baby crying?” Yes/No/Do not know and (3) Crying intensity, “What colour is your baby’s cry at the present moment?” Blue/Yellow/Red, where blue indicated a fussy cry, red indicated inconsolable crying and yellow indicated everything in between. Responses were coded 0 ‘No’, 1 ‘Yes’. Crying intensity was coded 0 ‘Blue’, 1 ‘Yellow’ and 2 ‘Red’. Higher scores indicated higher levels of distress. ‘Do not know’ responses were used for descriptive statistics only.

#### Maternal mood

The Edinburgh Postnatal Depression Scale (EPDS) (Cox et al. 1987) screens for depressive symptoms in the antenatal and postnatal period. The EPDS is a 10-item self-report measure on a 4-point scale from 0 to 3, with a score of 13 indicating severe depression and has shown satisfactory internal consistency (α = 0.87) (Cox et al. 1987).

#### Objective infant distress

Saliva sampling at home provided a degree of ecological validity of infant stress levels during a bedtime stressor. Based on Gunnar et al. (1996), samples were taken after breastfeeding and before the bedtime intervention began (baseline) and 45 min after starting the BSI with the infant in their sleep space. Salivary cortisol samples were gathered via oral swab (Salimetrics SalivaBio children’s swabs, SCS), by mothers placing an end of the SCS in their infant’s mouth, allowing pooled saliva to be absorbed onto the swab for 90 s. The swab was stored securely in the collection tube in the family refrigerator until collected. Saliva samples (25 μl) were assayed in duplicate using a highly-sensitive enzyme immunoassay (Stratech Scientific APAC PTY Ltd, NSW) with a range of sensitivity from 0.003 to 3.0 μg/dl (average intra- and inter-assay coefficients of variation were 4.5% and 4.9%, respectively). Higher cortisol concentrations indicate higher levels of infant stress.

#### Sleep diary

Completed four times (Table 1), a previously utilised sleep diary (Blunden & Bails 2013; Boutzious et al. 2014) gathered data on infant: time into bed; number of night-wakings (WASO), total minutes asleep overnight.

**Table 1:** Measures completed and sample sizes for each time point

Time point	Measures	Responsive *n*	CC *n*	Control *n*
T1 (pre-intervention, 1 week prior to starting)	Infant sleep Maternal EPDS	15	18	8
T2 First week of intervention	Infant sleep Maternal EPDS Maternal SUDS MPI-stress Cortisol	14	9	8
T3 2 weeks after T2	Infant sleep Maternal EPDS MPI-Stress Maternal SUDS	13	8	8
T4 4–6 weeks after T2	Infant sleep Maternal EPDS MPI-stress Maternal SUDS	13	8	8

### Procedure

After randomisation, all participants were given a sleep psychoeducation session, written instructions on protocols, SUDS and coding infant crying intensity and saliva sample collection protocol and sample collection demonstration (two intervention groups only).

On two consecutive evenings (day 1 and 2) participants were instructed to conduct their infant bedtime routine with questionnaires, followed by infant saliva sample collection (only twice). The first time (pre-stressor) occurred after feeding and prior to onset of bedtime routine (~ 6:30 pm) to standardise circadian timing and avoid elevated cortisol readings due to milk/food contamination (Schwartz et al. 1998). Then the infant was placed alone in their cot (i.e. onset of the bedtime separation stressor). The second time-point (post-stressor) occurred 40 min after placing the infant in bed (Gunnar et al. 1996). Saliva samples were forwarded to the research laboratory for analysis.

### Data analysis

All data were normally distributed except cortisol and were analysed with Intention-to-Treat principles. Paired-sample *t*-tests or Wilcoxon Signed Rank tested differences in maternal SUDS, maternal depression and infant salivary levels pre- and post-bedtime stressor. Point-biserial correlations evaluated the relationship between maternally perceived infant stress and infant physiological distress (cortisol levels). Multivariate ANOVA general linear models were utilised to evaluate differences in sleep duration and EPDS between groups. Following established protocols (Middlemiss et al. 2012), saliva samples (*n* = 5) with cortisol values considered biologically implausible (i.e. > 2.0 µg/dL) and three samples with insufficient volume were excluded from analysis. Raw SUDS scores and cortisol values were subjected to natural log transformations (Middlemiss et al. 2012), but raw values were reported to allow comparisons across studies. Partial eta squared (ɲp2) were calculated for sleep duration, depression scores and cortisol. Cortisol data was abnormally distributed so raw scores were subjected to a log transformation (LG10 + 1) to correct positive skew (Watamura et al. 2003). Cortisol was measured with a mixed model analysis undertaken with random effects of participant ID considered one between groups factor (treatment groups × three) and two within-subjects factors: Time with two levels (Before stressor and after stressor) and Day with two levels (day 1 and day 2). The mixed model analyses were chosen as they take into account different sources of variation in the data including standard deviations and age and accounts for correlations between scores taken close together (e.g. 40 min apart). Significance was set at *p* < 0.05, except for correlations (*p* = 0.02).

## Results

### Demographics

In total, 31 mother-infant dyads started the programme (demographic data available for 20 dyads). Data revealed that mothers were well-educated, all had partners and the majority perceived that their child had a sleep problem. Participants demographic data did not significantly differ from the original online survey sample (see Table 2).

**Table 2:** Demographic data (*n* = 20)

Variable	Unit of measurement
Mean (SD) infant age in months	9.55 (2.0)
Range infant age in months	6.6–12.4
% female infants	52%
% male infants	48%
Maternal marital status	100% married/defacto
English spoken at home	100%
Maternal age range in years	
21–25	0.5%
26–30	19.5%
31–35	60.0%
36–40	15.0%
Maternal education	
High school or diploma	19.0%
Undergraduate	57.1%
Higher tertiary education	19.0%
Severity of sleep problem rating	
Mother perceives a small sleep problem	75.0%
Mother perceives a serious sleep problem	25.0%
Ethnicity	Unknown

### Sleep patterns

Multivariate ANOVA found increases in sleep duration across all groups from T1–T4. Controlled Crying increased by 25 min compared to 12 for Responsive and 7 for Controls. However, these increases were not significant overall *F* (8, 28) = 1.63, *p* = 0.16; Wilk’s Λ = 0.47, partial n2 = 0.32 (see Table 3). So overall, the effect of intervention on sleep was not significant over time. However, at T3, Responsive reported significantly less sleep than other groups (*F (2, 22)* = 4.49 (*p* = 0.03) η_p_^2^ = 0.35 — large effect). This significant result did not effect the interaction between intervention and time overall.

**Table 3:** Mean , SD, significance and effect sizes for sleep duration (hours and minutes) across four time points (hours and minutes)

Variable	*Mean T1*	*SD T1*	*M T2*	*SD T2*	*M T3*	*SD T3*	*M T4*	*SD T4*
n	*R* = *11; CC* = *7; C* = *7*		*R* = *11; CC* = *7; C* = *7*		*R* = *9; CC* = *6; C* = *6*		*R* = *10; CC* = *6; C* = *6*	
Responsive (R)	10.28	1.03	10.27	0.19	10.20	1.01	10.36	1.07
Controlled Crying (CC)	10.16	0.33	10.19	1.03	11.01	0.52	11.01	1.00
Controls (C)	10.35	0.16	11.23	0.28	10.58	0.23	11.04	1.06
Between groups differences (ANOVA)	F (2, 22) = 0.554 (*p* = 0.59)		F (2, 22) = 1.96 (*p* = 0.17)		F (2, 22) = 4.80 (*p* = 0.02)		F (2, 22) = 0.01 (*p* = 0.99)	
Between groups effect size	ηp2 = 0.12 (Medium effect)		ηp2 = 0.19 (Large effect)		ηp2 = 0.35 (Large effect)		ηp2 = 0.01 (Small effect)	

NB:T1, Baseline; T2, 2 weeks post intervention; T3, 4 weeks post intervention; T4, 8 weeks post intervention

**Fig. 2:**
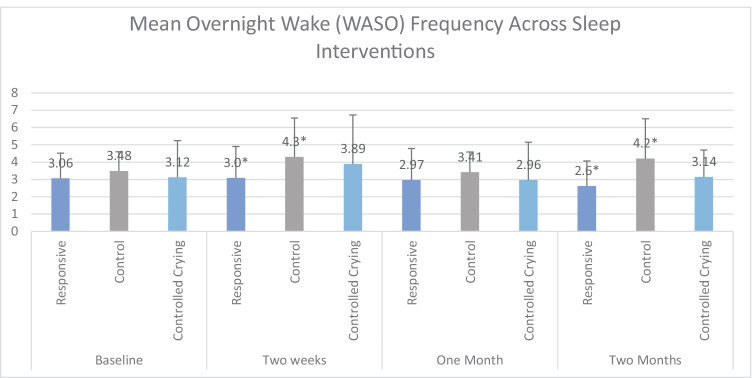
Mean number of awakenings with standard deviation bars. *Indicates significant differences between groups NB. WASO = Wake after Sleep Onset

Multivariate analysis showed no significant differences in the average number of wakes between groups at T1 and T3. There was a significant difference at T2 and T4 with Responsive recording the lowest WASO; *F* (2, 104) = 5.07, *p* = 0.008) and *F* (2, 99) = 6.416, *p* = 0.002, respectively. The Controlled Crying group did not change across the 4 timepoints (*p* > 0.05*)* and Controls were significantly higher than both other groups at T4 (*F* (2, 99) = 6.416, *p* = 0.002) (see Fig. 2).

### Maternal depression

**Fig. 3:**
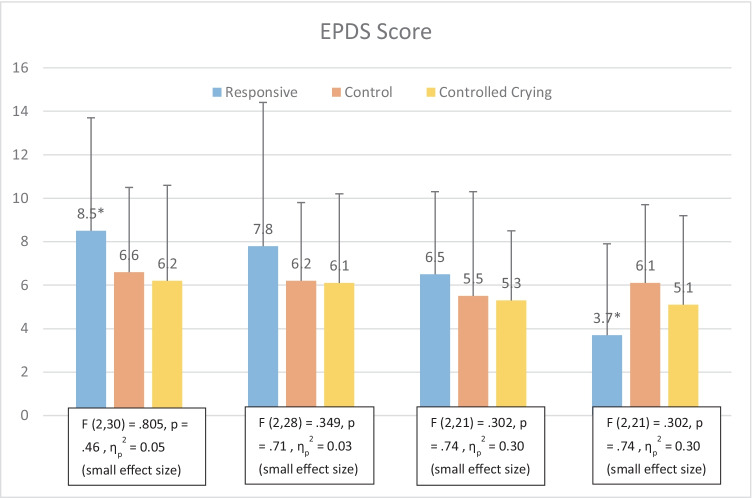
Visual representation of EPDS scores across time points and results of Multivariate ANOVA. *indicates sigificant differences between T1 and T4 for Responsive group. Scores < 8 are considered in normal range

None of the participants reported clinically significant levels of depression on the EPDS (e.g. > 13 (see Fig. 3). At T2 and T4, Controls’ Maternal EPDS scores were higher than Responsive. Responsive EPDS significantly decreased between T1 (EPDS, T1 = 8.54 (SD 5.22) and T4, (EPDS T4 = 3.75 (SD 4.23), (*t*(20) = 2.14, *p* = 0.04) (see Fig. 3). However, Multivariate ANOVA showed no significant differences in EPDS scores *F* (8, 28) = 0.57, *p* = 0.79; Wilk’s Λ = 0.74, partial n2 = 0.14 across intervention groups. So overall, the effect of intervention on depression was not significant over time. Results are presented in Table 4.

**Table 4:** Subjective infant distress measurements pre- and post- bedtime stressor

	Pre-stressor:* n* (%)	Post-stressor:* n* (%)	χ^2^: *p value*
Day 1			
Infant stressed?			
No	15 (93.8)	12 (75)	.333*
Yes	1 (6.3)	3 (18.8)	
Do not know^b^	0	1 (6.3)	
Infant crying?			
No	15 (93.8)	12 (75)	.333*
Yes	1 (6.3)	4 (25)	
Do not know^b^	0	0	
Crying intensity^a^			
Not crying**^b^	11 (68.8)	8 (50)	.999^a^
Fussy cry	5 (31.3)	6 (37.5)	
Intermediate cry	0	1 (6.3)	
Inconsolable cry	0	1 (6.3)	
Day 2			
Infant stressed			
No	14 (87.5)	13 (81.3)	.999*
Yes	1 (6.3)	2 (12.5)	
Do not know^b^	1 (6.3)	1 (6.3)	
Infant crying			
No	14 (87.5)	12 (75)	.654*
Yes	2 (12.5)	4 (25)	
Do not know^b^	0	0	
Crying intensity^a^			
Not crying** ^b^	10 (62.5)	9 (56.3)	.999^a^
Fussy cry	6 (37.5)	6 (37.5)	
Intermediate cry	0	1 (6.3)	
Inconsolable cry	0	0	

*Fisher’s Exact Test use for analysis due to low cell frequencies. ** ‘Not crying’ is representative of infants not reported as having a crying intensity

^a^Fisher’s Exact test was performed on pooled Intermediate and Inconsolable categories compared to fussy cry category. ^b^Excluded from Fisher’s Exact test analysis

### Stress

#### Maternal and infant stress

Paired-sample *t*-tests showed maternally perceived infant stress, infant crying, crying intensity and cortisol levels increased between pre- and post- stressor (see Table 4) but these did not reach significance. Due to low (i.e. < 5) cell frequencies for ‘crying intensity’, a 2 × 2 Fisher’s Exact Test was performed on pooled data comparing ‘Fussy cry’ and combined ‘Intermediate’ and ‘Intolerable’ crying intensities (see Table 4).

#### Maternal distress (SUDS) and maternal perceived infant stress (MPI-S)

ANOVA were conducted on mean SUDS scores taken post bedtime separation across all four time points. There were no respondents in the Controlled Crying group at T3 and only 1 at T4. Means SUDS at T1 and T2 were higher in Controlled Crying, compared to the other two groups which was significant at T1 (*p* = 0.01*)* and showed a trend at T2 (*p* = 0.057) (Table 5).

**Table 5:** Mean (SD) SUDS scores

Group	Mean (SD) T1	Mean (SD) T2	Mean (SD) T3	Mean (SD) T4
Responsive *N* = 13	21.10 (1.9)	18.92 (3.2)	17.29 (4.7)	17.08 (4.8)
Controlled Crying *N* = 9	29.01 (4.7)*	31.34 (15.9)	No data	13.33 (7.6)
Controls *N* = 8	16.00 (3.7)	14.18 (4.4)	10.60 (5.5)	16.4 (5.5)

NB* indicates significant at *p* = 0.01

#### Maternal SUDS stress relative to MPI-S in the whole sample (*n* = 15)

Point-biserial correlations found the relationship between maternal SUDS and perceived infant stress (MPI-S) were not significant but showed trends post stressor (adjusted α = 0.027) on day 1, with a similar finding (*p* = 0.027), pre stressor day 2 (see Table 6).

**Table 6:** Point-biserial correlation between maternal SUDS and MPI-S pre- and post- bedtime stressor

	*N*	*r*_*pb*_	*Size and direction of relationship*	*(p)*
Day 1				
Pre-stressor				
SUDS and MPI-S	15	.462	Moderate positive	.063
SUDS and infant crying	15	.462	Moderate positive	.063
Post-stressor				
SUDS and MPI-S	15	.300	Weak positive	.278
SUDS and infant crying	15	.469	Moderate positive	.027
Day 2				
Pre-stressor				
SUDS and MPI-S	14	.588*	Moderate positive	.027*
SUDS and infant crying	14	.272*	Weak positive	.347*
Post-stressor				
SUDS and MPI-S	15	.466*	Moderate positive	.080*
SUDS and infant crying	15	.484*	Moderate positive	.067*

*Due to normality violations for day 2 pre- and post-stressor SUDS data, a Spearman’s rho was used for the analysis. Note: An adjusted alpha of 0.025 was used to correct for the Type I familywise error rate

#### Objective stress — cortisol

**Fig. 4:**
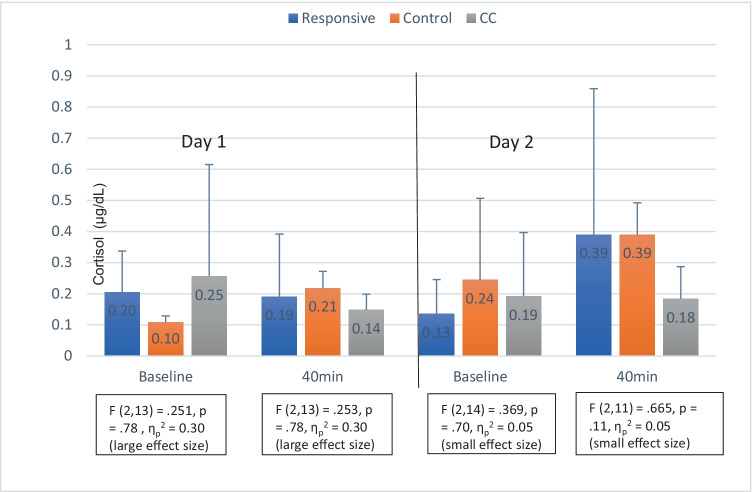
Cortisol levels during 2 days of collection across groups and results of multivariate ANOVA — Responsive (*n* = 7), Controlled Crying (CC) (*n* = 6) and Control (*n* = 4)

Raw cortisol values taken 40 min after the start of the bedtime stressor were comparable between groups (Fig. 4). Multivariate ANOVA confirmed no significant differences between groups or within groups at any time point nor changes over time.

Linear mixed models analysis showed no effect for condition — Treatment Group (F (2, 40) = 0.63, *p* = 0.54); dependent variable 1 — Pre vs Post stressor (F (1, 40) = 0.13, *p* = 0.71) or dependent variable 2 — Day (F (1, 40) = 0.01, *p* = 0.92). There were no interaction effects (all *p* < 0.05). There was significant variability in the cortisol levels both inter and intra individually, with several age groups clustered together.

## Discussion

For many years, mothers have expressed concern about the impact of infant crying at the moment of separation during BSI that involve extinction. Findings from this pilot RCT are the first to evaluate whether a responsive BSI is comparable to an extinction method by measuring objective (infant) and subjective (maternal and maternally reported) stress, maternal depressive symptomology and infant sleep. The small sample showed no significant differences in cortisol nor sleep duration between groups, although the number of overnight wakings significantly decreased in the Responsive group only. Nonetheless, mothers correctly identified stress in their child and maternal stress and symptoms of depression were reduced in the Responsive group compared to the other groups. This suggests that when mothers are able to attend to their distressed child, the emotional impact on their mood and stress is reduced. However, future studies need to replicate these findings in larger more diverse samples to confirm if Responsive, non-ignoring methods are acceptable to mothers and achieve similar outcomes to extinction techniques, therefore offering increased intervention choices for families of sleep disturbed infants.

For sleep, there were no overall differences in sleep duration suggesting Responsive and Controlled Crying are equally effective in ameliorating infant sleep disturbance. This confirms previous findings of the same responsive protocol (Blunden and Bails 2013; Boutzious et al. 2014) but adding reduced night-wakings in the Responsive group.

The cortisol levels, measured *at the time of the bedtime stressor*, did not differ between groups. Previous studies have attempted to show that leaving a child to cry does not impact cortisol levels but cortisol was rarely measured at the time of separation but hours (Gradisar et al. 2016), or days (Gradisar et al. 2016; Cook et al. 2012), afterwards. So, not only was the stress during separation stressor not evaluated, but there are many factors that can impact cortisol and stress between extinction execution and cortisol collection. Our data showed no significant difference between groups (albeit small groups), suggesting a crying infant left alone shows similar distress levels to a child given a reduced level of bedtime interaction (such as not rocking or feeding to sleep). Indeed Kahn et al. (2019) note that all behavioural interventions where parents modify soothing behaviours typically involve some level of infant crying at first. However, this study *was* able to demonstrate that mothers correctly identified when the infants were stressed with increased crying increasing maternal stress. So, any separation between mother and child is stressful but when mothers were able to attend and respond to the stressed child, mothers perceived less stress in their infant, reported less stress themselves, and fewer symptoms of depression compared to other groups. In effect, this may be one of the important findings from this study perhaps suggesting gentle treatment approaches may be efficacious and acceptable. It is important to note that attrition rates were greater from the Controlled Crying group in this study and this is consistent with previous randomised studies (Gradisar et al. 2016) showing parental reticence to utilise extinction which appear to collide with some parenting styles. So, instead of trying to measure stress levels during a bedtime separation, to “prove” (or disprove) infant distress, listening to the voices of the mothers and the crying of infants whose stress is evident at the moment of separation is informative and important. Indeed, maternal reticence to utilise extinction has persisted, with or without the evidence regarding cortisol levels.

However, cautionary interpretation of these data is necessary. Small and irregular sample sizes, exacerbated by attrition for certain time points and questionnaires, with mostly self-report data suggest findings should be interpreted with caution. Despite the knowledge that cortisol levels are unreliable in young infants becoming more reliable with age (Thompson et al. 2015), the age range and small sample size disallowed controlling for age. So, cortisol reactivity may not be a sensitive indicator (Jansen et al. 2010; Gunnar et al. 2009). Another factor unexplored in this study was infant fussiness and maternal temperament (e.g. anxiety and cognitions about crying). These factors have been implicated in the mother/infant dyad sleep patterns and impact how mothers navigate sleep interventions (Sadeh et al. 2010; Tikotzky 2017; Tikotzky et al. 2021). Finally, this pilot study did not explore culture and ethnicity of the participating families. Given that sleeping arrangements and parenting are impacted by these factors (Schwichtenberg et al. 2019), their impact on sleep and settling interventions, including perceptions and uptake of (or indeed attrition from) sleep interventions, should be further explored. Future studies should include measures of infant separation anxiety and maternal temperament in larger sample sizes.

Despite these limitations, this study adds to the literature on responsive sleep interventions in young children and maternal/infant mental health. The important issues in question, sleep consolidation vs stress levels in crying infants and subsequent maternal stress levels, must be further explored in future larger more comprehensive studies, given the documented need by mothers for more responsive BSI. To date, published empirical data for Responsive BSI are limited so this pilot study is a step towards this and future research in this area will increase the choice offered to parents who wish to improve their infant’s sleep but do not wish to ignore them.

## Data Availability

All data is available on request and was available to all authors.
